# The effectiveness of Chinese herbal medicine for tic disorders in children and adolescents: A protocol for systematic review and meta analysis

**DOI:** 10.1097/MD.0000000000028190

**Published:** 2021-12-23

**Authors:** Li Liu, Liang-An Zhou, Ya-Lei Sun

**Affiliations:** a Department of Chinese Medicine, Jiaxing Maternity and Child Health Care Hospital, No. 2468 of Central East Road, Nanhu District, Jiaxing, Zhejiang, China; b Intensive Care Unit, Pinghu Hospital of Traditional Chinese Medicine, No. 74 Danghu West Road, Pinghu, Zhejiang, China; c Affiliated Hospital of Nanjing University of Chinese Medicine, No. 155 Hanzhong Road, Qinhuai District, Nanjing, Jiangsu, China.

**Keywords:** Chinese herbal medicine, meta-analysis, protocol, randomized controlled trials, tic disorders

## Abstract

**Background:**

The tic disorders are common neuropsychiatric disorders that affects the growth and development of children and adolescents. Chinese herbal medicine is commonly used for the treatment of tic disorders. However, there is no consensus on the difference in clinical efficacy compared with routine treatment. Therefore, we plan to perform a systematic review and meta-analysis to review the clinical efficacy of Chinese herbal medicine for tic disorders.

**Methods:**

Cochrane Central Register of Controlled Trials, PubMed, EMBASE, Chinese National Knowledge Infrastructure, Chinese Bio-medical Database, and Wanfang database will be searched from their inception until March 31, 2021. The meta-analysis will be conducted with Review Manager 5.3 software to systematically review the clinical efficacy and safety of Chinese herbal medicine for tic disorders. The primary outcome will include the improvement rate (amount) of tic symptoms using related scales or methods, and the secondary outcome will include adverse events.

**Results:**

This analysis will provide useful information about clinical efficacy and safety of Chinese herbal medicine for tic disorders.

**Conclusions:**

Our study will generate strong evidence of Chinese herbal medicine for patients with tic disorders and provide suggestions for clinical practice.

## 1 Introduction

Tic disorders, including transient tic disorder (TTD), chronic motor or vocal tic disorder (CTD), and Tourette syndrome (TS), are common neuropsychiatric disorders among children and adolescents. Tic disorders, which are characterized by repetitive, sudden, nonrhythmic vocalization or motor movement, are observed in approximately 6% to 20% of children worldwide.^[1]^ A meta-analysis of the prevalence of tic disorders showed that globally, the prevalence of TTD is 2.99% (95% confidence interval [CI]: 1.60-5.61), CTD has a prevalence of 1.61% (95% CI: 0.92-2.83), and TS has a prevalence of 0.77% (95% CI: 0.39-1.51).^[2]^ The prevalence of TTD, CTD, and TS in China are 1.7% (95% CI: 0.009-0.031), 1.2% (95% CI: 0.007-0.022) and 0.3% (95% CI: 0.001-0.008), respectively.^[3]^ These results show that TTD is the most common type of tic disorder in children, followed by CTD and TS.

At present, haloperidol, risperidone, aripiprazole, tiapride, and clonidine are become commonly employed treating tic disorders. However, due to the chronic nature of tic disorders, drug treatment is usually long-term. Long-term drug treatment will bring a series of adverse drug reactions to patients, including weight gain, drug-induced movement disorders, elevated prolactin levels, sedation, and effects on heart rate, blood pressure, and electrocardiograms.^[4]^

In consideration of its limitations, the application of Chinese herbal medicine could be promoted. In traditional Chinese medicine (TCM) theories, the pathogenesis of tic disorders is “agitation of liver wind, lending to excessive wind and spasm of tendons”. Consequently, the core of treating is to balance the yin and yang of “meridian tendons” and regulate spirit.^[5]^ In recent years, randomized controlled trials (RCTs) on the treatment of tic disorders with Chinese herbal medicine have been reported. However, compared with routine treatment, there is no consensus on the difference in clinical efficacy. Therefore, we plan to conduct this meta-analysis to systematically review the clinical efficacy of Chinese herbal medicine for tic disorders. This analysis is expected to obtain meaningful conclusions and provide a high level of evidence in evidence-based medicine.

## 2 Methods

### 2.1 Study registration

The protocol for this meta analysis was registered at PROSPERO with the registration number CRD42019135168 (URL=https://www.crd.york.ac.uk/prospero/display_record.php?RecordID=135168). The preferred reporting items for systematic review and meta-analysis protocols statement checklist for reporting meta-analyses was used in this study.^[6]^

### 2.2 Inclusion criteria

All of the inclusion criteria in the population/intervention/comparison/outcomes/study design order will be met by the studies included in this meta-analysis.

#### 2.2.1 Types of patients

The study will include children and adolescents patients were diagnosed with tic disorders. The age of participants was younger than 18 years. The widely used definitions of tic disorders are in the following guidelines: the Diagnostic and Statistical Manual of Mental Disorders-III (DSM-III), DSM-IV, or DSM-IVText Revision^[7–9]^; the International Classification of Diseases-10 (ICD-10)^[10]^; and the Chinese Classification and Diagnostic Criteria of Mental Disorders (CCMD).^[11]^ There were no restrictions on sex, course of disease, or course of treatment.

#### 2.2.2 Types of interventions

The treatment group received Chinese herbal medicine, and the control group received conventional treatment (e.g., aripiprazole, haloperidol, tiapride, ziprasidone, olanzapine and so forth).

#### 2.2.3 Types of outcome

The outcomes of the efficacy were assessed based on the following standard tools: The Yale Global Tic Severity Scale (YGTSS); The Tourette Syndrome Global Scale (TSGS); The Tourette Syndrome Symptom List; The Clinical Global Impression Scale (CGI); The Tourette Syndrome Severity Scale (TSSS); and The Clinical Global Impression Tic Severity Scale.^[8,12–14]^ Adverse events (AEs) were assessed by the Treatment Emergent Symptom Scale (TESS).^[15]^

#### 2.2.4 Types of studies

RCTs using Chinese herbal medicine to treat tic disorders regardless of blinding or allocation concealment.

### 2.3 Search strategy

We will perform a systematic search in both English and Chinese databases from their inception to March 31, 2021: Cochrane Central Register of Controlled Trials, PubMed, EMBASE, Chinese National Knowledge Infrastructure, Chinese Bio-medical Database, and Wanfang database. There are no language restrictions. The medical subject headings and free text words will be applied. The following 3 terms will be used as the search strategy and modified to suit each database: health condition (tic disorders), Chinese herbal medicine, study type (randomized controlled trials). We will simply provide the search process of the PubMed and EMBASE (Tables 1 and 2). In addition, reference lists of identified papers will also be checked for additional papers.

**Table 1:** PubMed search strategy.

No.	Search terms
# 1	(((((((((Tic Disorders[MeSH Terms]) OR (Tic Disorders[Title/Abstract])) OR (Chronic Motor Vocal Tic Disorders[Title/Abstract])) OR (Transient Tic Disorders[Title/Abstract])) OR (Post-Traumatic Tic Disorders[Title/Abstract])) OR (Vocal Tic Disorders[Title/Abstract])) OR (Motor Tic Disorders[Title/Abstract])) OR (Tourette Syndrome[Title/Abstract])) OR (Tourette Disease[Title/Abstract])) OR (Tourette Disorder[Title/Abstract])
# 2	((((((Medicine,Chinese Traditional[MeSH Terms]) OR (Chinese Medicine[Title/Abstract])) OR (Chinese Herbal Medicine[Title/Abstract])) OR (Herbal Medicine[Title/Abstract])) OR (Medicine, Herbal[Title/Abstract])) OR (zhongyi[Title/Abstract])) OR (zhongyao[Title/Abstract])
# 3	((((((((randomized controlled trial[Publication Type]) OR (controlled clinical trial[Publication Type])) OR (randomized[Title/Abstract])) OR (randomly[Title/Abstract])) OR (placebo[Title/Abstract])) OR (trial[Title/Abstract])) OR (groups[Title/Abstract])) OR (drug therapy[MeSH Terms])) OR (drug therapy[MeSH Subheading])
# 4	#1 AND #2 AND #3

**Table 2:** EMBASE search strategy.

No.	Search terms
#1	(“Tic Disorders” /exp) OR (“Tic Disorders”:ti,ab,kw) OR (“Chronic Motor Vocal Tic Disorders”:ti,ab,kw)OR (“Transient Tic Disorders”:ti,ab,kw) OR (“Post-Traumatic Tic Disorders”:ti,ab,kw) OR (“Vocal Tic Disorders”:ti,ab,kw) OR (“Motor Tic Disorders”:ti,ab,kw) OR (“Tourette Syndrome”:ti,ab,kw) OR (“Tourette Disease”:ti,ab,kw) OR (“Tourette Disorder”:ti,ab,kw)
#2	(“Chinese Medicine”/exp) OR (“Traditional Chinese Medicine”:ti,ab,kw) OR (“Chinese Herbal Medicine”:ti,ab,kw) OR (“Herbal Medicine”:ti,ab,kw) OR (“Medicine,Chinese Traditional”:ti,ab,kw) OR (“Medicine, Herbal”:ti,ab,kw) OR (“zhongyi”:ti,ab,kw) OR (“zhongyao”:ti,ab,kw)
#3	(“randomized controlled trial”/exp OR “randomized controlled trial”) OR (“controlled clinical trial”/exp OR “controlled clinical trial”) OR (“randomized”:ti,ab,kw) OR (“controlled”:ti,ab,kw) OR (“trial”:ti,ab,kw) OR (“random”:ti,ab,kw) OR (“group”:ti,ab,kw)
#4	#1 AND #2 AND #3

### 2.4 Study selection and data extraction

**Figure 1:**
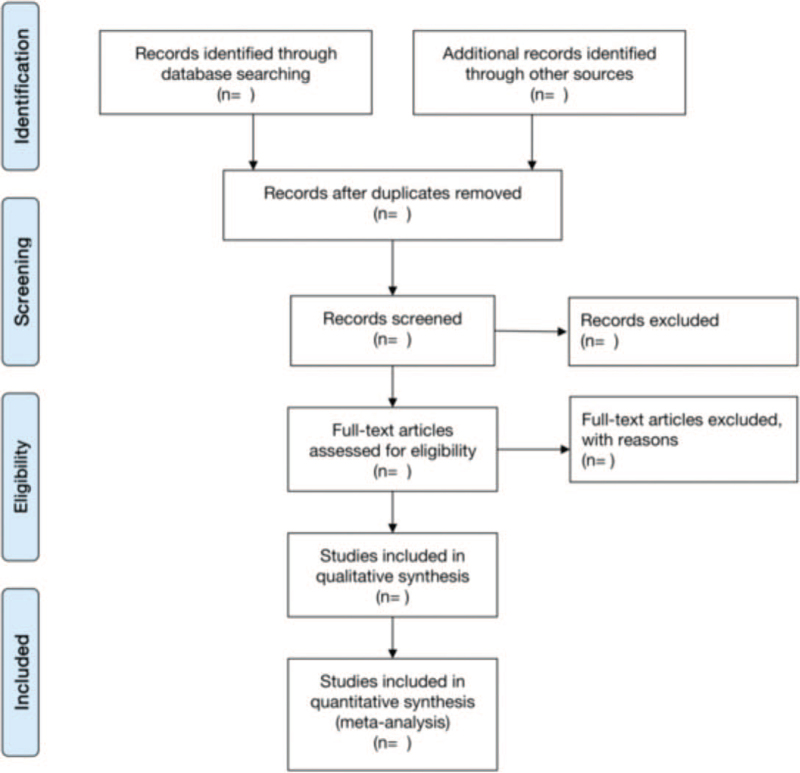
Flow diagram of the study search.

Retrieved papers will be managed by NoteExpress 3.0 software (Beijing AQHYZ Technology Co., Ltd. Beijing, China.). Two review authors independently read the tittles and abstracts to exclude the wrong types of literature (e.g., irrelevant, review, animal experiment). The remaining literature will be read in full text to identify any eligible studies. The data extraction items include first author, publication date, sample size, course of disease, interventions, course of treatment, outcomes. Any disagreement will be resolved by discussion with a third review author. The selection process will be provided in Figure 1.

### 2.5 Risk of bias assessment

The methodological quality of the eligible studies will be evaluated by 2 review authors according to the Cochrane Handbook for Systematic Reviews of Interventions.^[16]^ The details include random sequence generation, allocation concealment, blinding of participants and personnel, blinding of outcome assessors, incomplete outcome data, selective reporting and other bias. Each entry will be assessed as “low risk”, “unclear risk” or “high risk”. Any disagreement will be resolved by discussion with a third review author.

### 2.6 Statistical analysis

Cochrane Collaboration's Review Manager 5.3 software will be used to perform the meta-analysis. In general, conducting a meta-analysis requires at least 2 studies reporting the same outcome variables. If 2 or more homogeneous studies are available, we will use aggregated data for meta-analysis. To perform meta-analysis of dichotomous data, the meta-analysis need the rate (or ratio) in order to pool data. For dichotomous outcomes, we will use the relative risk, with 95% CI and *P* values. To perform meta-analysis of continuous data, the meta-analysis need the mean value and standard deviation in order to pool data. Continuous variable will be described by mean difference, *P* value and 95% CI. Heterogeneity will be assessed using the *I*^2^. *I*^2^ ≤ 50% indicate that the studies have homogeneity, so fixed effects model will be used, otherwise the random effects model will employed for analysis.^[17]^ Subgroup analysis for outcomes will be performed based on prespecified effect modifiers as follows: study quality, sample size, age, gender, treatment duration, etc. If the data are not available for quantitative analysis, we will report result by qualitative description. If adequate trials are included in the study (>10 trials), funnel plot and Egger test will be performed to detect publication bias. When the *P* < .05 and the funnel plot is asymmetric, publication bias is considered to be present.

### 2.7 Quality of evidence

Based on the grades of recommendations assessment, development, and evaluation system, evidence quality and recommendation levels will be evaluated. The quality-assessment domains including downgrade quality of evidence (risk of bias, inconsistency, indirectness, imprecision and publication bias) and upgrade quality of evidence (large effect, dose-response gradient and plausible confounding).^[18]^

## 3 Discussion

Tic disorders are a group neuropsychiatric disorders with childhood onset characterized by tic. Some studies have estimated that about 60% to 80% of children with tic disorders experience symptoms that can last until the age of 16 years, and about 23% of adolescents experience moderate and severe tics, which seriously affect social functioning.^[19]^ For example, elevated levels of peer victimization, social deficits and psychiatric comorbidity including depressive symptoms, mood disorders, disruptive behavior disorders, and attention/hyperactivity problems have been documented in youth with tic disorders.^[20,21]^

The main etiologies of tic disorders are genetic and environmental factors. Epidemiological studies have shown that the heritability of tic disorders is between 28% to 56%.^[22]^ And in terms of environmental factors, maternal smoking, prenatal life stressors, lower birth weight, and A streptococcal infections appears to correlate with tic severity.^[23]^ The pathogenesis of tic disorders could be due to a combination of genetic, immunological, psychological, and environmental factors. The links between the pathophysiology and clinical symptoms probably lie in the disinhibition of the cortical-striatum-thalamus-cortical circuits. An imbalance of inhibitory-excitatory signals in these circuits is considered as the molecular mechanism to produce the tics and related symptoms. As reported, tics is related to an underlying dysfunction of corticostriatothalamocortical circuits. Tics have been interpreted as the result of a focal excitatory abnormality in the striatum.^[24]^ Tics also seems associated with dysfunction in different neurotransmitter systems.^[25]^ Hyperdopaminergic tone seems to be the most relevant neurochemical abnormality in tics. An overactive dopamine transmission, particularly striatal, could cause excessive reinforcement of learned motor sequences, which can lead to tic.^[26]^

Antipsychotic drugs are commonly used in the treatment of tic disorders, including typical antipsychotic drugs (such as haloperidol, pimozide, etc) and atypical antipsychotic drugs (such as risperidone, aripiprazole, quetiapine, etc). However, these drug treatment will bring a series of adverse drug reactions to patients. In a network meta-analysis involving 60 RCTs (4077 participants), 57 studies (95%) reported the occurrence of adverse reactions. The most common adverse events were drowsiness, extrapyramidal reactions and nausea/vomiting.^[27]^

In recent years, the RCTs of Chinese herbal medicine for the treatment of tic disorders have gradually increased. Numerous literature has suggested that the application of Chinese herbal medicine holds a significant position for tic disorders. However, the difference in clinical efficacy compared with routine treatment is uncertain. This meta-analysis will be the first review to review the effectiveness of Chinese herbal medicine for the treatment of tic disorders. We hope that the results of our study will provide the clinical recommendation for patients with tic disorders, and promote the level of evidence-based medicine in Chinese medicine.

## Author contributions

**Conceptualization:** Li Liu, Ya-Lei Sun.

**Formal analysis:** Li Liu.

**Methodology:** Liang-An Zhou.

**Writing – original draft:** Li Liu, Liang-An Zhou, Ya-Lei Sun.

**Writing – review & editing:** Li Liu.
